# New reduced-risk agricultural nematicides - rationale and review

**DOI:** 10.21307/jofnem-2020-091

**Published:** 2020-09-03

**Authors:** Johan Desaeger, Catherine Wram, Inga Zasada

**Affiliations:** 1 Department of Entomology and Nematology, University of Florida, 14625 CR 672, Wimauma, FL, 33598; 2 Department of Botany and Plant Pathology, Oregon State University, Corvallis, OR, 97331; 3 USDA-ARS, Horticultural Crops Research Laboratory, 3420 NW Orchard Avenue, Corvallis, OR, 97330

**Keywords:** Efficacy, Fluazaindolizine, Fluensulfone, Fluopyram, Plant-parasitic nematode

## Abstract

The last decade has seen a sharp increase in nematicide research in the agricultural industry. As a result, several new synthetic nematicides have become available to growers, and several more are expected in the near future. This new interest in nematicides is directly related to the growing demand for safer and more selective products, and the increasing regulatory pressure on many of the traditional nematicides. This has led to a ban of several widely used fumigant (e.g. methyl bromide) and non-fumigant (e.g. aldicarb) nematicides. The loss of traditional nematicides, combined with a lack of replacement products and awareness of the damage that nematodes can cause, has not only raised concern among growers, but has also created new opportunities for the crop protection industry. Nematicides have become a priority, and many companies are now allocating significant research dollars to discover new nematicides. The new nematicides are very different from previous products: (i) they are more selective, often only targeting nematodes, and (ii) they are less toxic, and safer to use. This review article describes these new developments by discussing the challenges that are associated with finding new nematicides, reviewing the nature, characteristics, and efficacy of new nematicides, and discussing the impact they could have on future nematode management.

Nematicides can be credited for having put the science of nematology firmly on the map. The enormous amount of crop damage and yield loss that plant-parasitic nematodes can cause was not known until the first trials with nematicides in the 1920s (Taylor, 2003). From the 1950s to the 1970s, the discipline of nematology was booming and research on nematode biology, physiology, and management was rapidly expanding. This optimism started changing in the 1960s and 1970s, when some of the less desirable side effects of nematicides started to emerge (Thrupp, 1991; Chitwood, 2003). This was primarily due to their high and broad-spectrum toxicity, and significant environmental impact (Chitwood, 2003). Unlike herbicides, insecticides, and fungicides, for which safer products have been available for decades, nematicides seem to have been stuck in the 1960s and 1970s, an age when regulatory requirements were in their infancy. Rachel Carson published ‘Silent Spring’ in 1962 (Carson, 1962), the first publication to raise awareness of the damage that pesticides can do to the environment, which eventually led to the establishment of the Environmental Protection Agency (EPA) in 1970 (Lewis, 1985). Almost all currently used nematicides predate the establishment of the EPA and would not pass the regulatory hurdles for new pesticides that are in place today. Traditionally, nematicides were broad-spectrum products, either fumigants (soil sterilants), or organophosphates or carbamates (neural toxins), and many of them have been banned in recent years. Several comprehensive reviews on nematicides have been written (Taylor, 2003, retroactively published in 2003; Wright, 1981; Hague and Gowen, 1987; Chitwood, 2003; Rich et al., 2004; Jones, 2017). A list of products that have been used as nematicides throughout history is given in Table 1.

**Table 1.** Products that have been used as nematicides throughout history.

Common name^a^	First use (country)	Product type/chemistry	Mode-of-action^b^	Signal words^c^
Carbon disulfide	1869 (FR)	Fumigant	Multi-site	Danger**
Chloropicrin	1920/1936	Fumigant	Multi-site	Danger
Methyl bromide	1932/1961	Fumigant	Multi-site	Danger*
Formaldehyde	1930	Fumigant	Multi-site	Danger**
DD	1943	Fumigant	Multi-site	Danger**
EDB	1945	Fumigant	Multi-site	Danger**
DBCP	1954	Fumigant	Multi-site	Danger**
1,3-D	1954	Fumigant	Multi-site	Danger
Metam sodium	1954	MIT generator	Multi-site	Danger
Fensulfothion	1957	Organophosphate	AChE	Danger**
Ethoprop	1963 (US)	Organophosphate	AChE	Danger
Aldicarb	1965 (US)	Carbamate	AChE	Danger*
Dazomet	1967	MIT generator	Multi-site	Danger
Carbofuran	1969	Carbamate	AChE	Danger*
Fenamiphos	1968 (DE)	Organophosphate	AChE	Danger*
Oxamyl	1972 (US)	Carbamate	AChE	Danger
Terbufos	1974 (US)	Organophosphate	AChE	Danger*
Enzone	1978	Fumigant	Multi-site	Danger*
Cadusafos	1990? (US)	Organophosphate	AChE	Danger*
Imicyafos	2010 (JPN)	Organophosphate	AChE	Danger*
Fosthiazate	1992 (JPN)	Organophosphate	AChE	Danger*
Ivermectin/Abamectin	1981 (JPN)	Lactone	GluCl	Danger
Spirotetramat	2008 (US)	Tetramic acid	LBI	Caution
DMDS	2010 (US)	Fumigant	Multi-site	Danger*
Methyl iodide	2007 (US)	Fumigant	Multi-site	Danger**
Allyl ITC	2013 (US)	Fumigant	Multi-site	Danger
Tioxazafen (seed)	2017 (US)	Oxadiazole	Unknown	Caution*
Fluensulfone	2014 (US)	Thizaole	Unknown	Caution
Fluopyram	2010 (US), 2013 (HND)	Benzamide	SDHI	Caution
Fluazaindolizine	2020?	Carboxamide	Unknown	Caution

**Notes:** ^a^DD = dichloropropane-dichloropropene mixture; EDB = ethylene dibromide; DBCP = 1,2-dibromo-3-chloropropane; 1,3-D =1,3-dichloropropene; Enzone = sodium tetrathiocarbonate (carbon disulfide liberator); MIT = methyl isothiocyanate generator; Allyl ITC = allyl isothiocyanate; DMDS = dimethyl disulfide; ^b^AChE = Acetylcholinesterase inhibitors; inhibition is reversible for carbamates, and irreversible for organophosphates; GluCl = Glutamate-gated chloride channel allosteric modulators; SDHI = succinate dehydrogenase inhibitors; LBI = Lipid Biosynthesis Inhibitor; ^c^*Limited registration; **no longer available.

**Sources:** Newhall (1955), Chitwood (2003); http://nemaplex.ucdavis.edu/Mangmnt/Chemical.htm; https://sitem.herts.ac.uk/aeru/iupac/Reports/19.htm.

The lack of nematicide research by industry from the 1960s to the last decade is in part due to the cryptic nature of nematodes, and the difficulty of recognizing and assessing impacts on crop yield, which often leads to an underestimation of the damage that they can cause. In addition, and probably the main reason for the research gap, is that the nematicide market is very small when compared with the herbicide, fungicide, and insecticide markets (Kang et al., 2016, Fig. 1). The entire history of nematicides in fact is one of accidental discoveries, as all of them were initially discovered not as nematicides, but rather as sterilants or fumigants (methyl bromide, 1,3-dichloropropene, metam), insecticides (oxamyl, ethoprop, and other organophosphates and carbamates), fungicides (fluopyram), or animal health drugs (abamectin).

**Figure 1:**
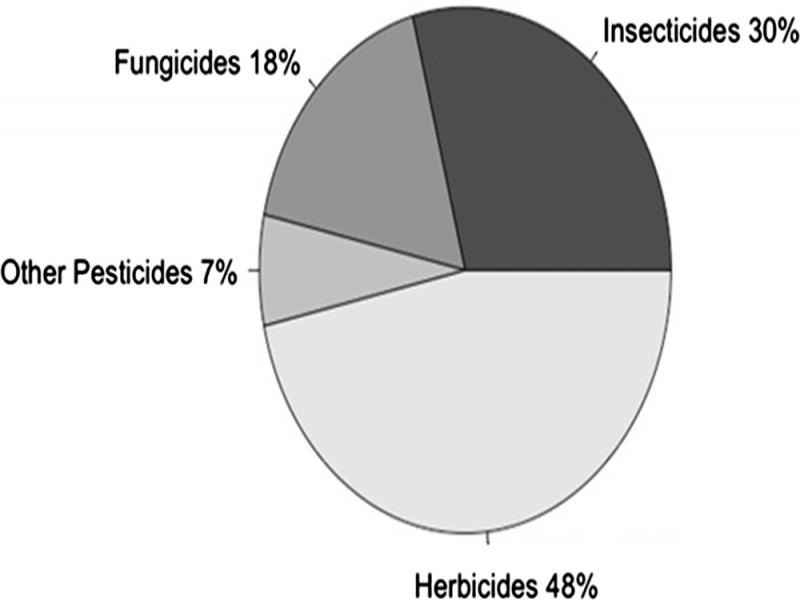
Global use of crop protection chemicals (Kang et al., 2016).

## Necessity is the mother of invention

The recent focus on nematicides is in large part a response to the overall increasing regulatory pressure on hazardous products (Class 1 pesticides, which until recently all nematicides belonged to), and more specifically the fact that some of the most effective and popular nematicides, including methyl bromide, fenamiphos, and aldicarb have become severely restricted (Ristaino and Thomas, 1997; Cone, 2010; EPA, 2010). These factors, combined with a growing awareness of the importance of managing nematodes in agriculture, and the expectation of more crop damage attributed to nematodes in the future due to agricultural intensification, soil degradation, and warmer climate, have triggered a new sense of urgency and opportunity within the agricultural industry.

A. L. Taylor wrote several publications on nematicides staring in the 1940s (Taylor and McBeth, 1940, 1941a, 1941b; Taylor, 1943, 1949). This was the time when first the fumigants and later the organophosphate and carbamate nematicides were introduced, and many fumigants were openly sold to the public in glass jugs (Nemagon, a.i. 1,2-dibromo-3-chloropropane; DBCP) or cans (Dowfume, a.i. methyl bromide). In one of his last papers from 1977 (retroactively published in 2003), Taylor (2003) ends with the following sentence: ‘During the course of an investigation started in 1977, the Environmental Protection Agency of the United States Government cited health hazards (‘groundwater contamination and male sterility’ note from author)… in manufacture, handling and application of DBCP… This event will certainly have a considerable influence on the future history of nematicides. Perhaps it is the beginning of a new era.’ DBCP, one of the most effective and widely used nematicides in history, was banned two years later (Babich et al., 1981). However, DBCP was effectively replaced by methyl bromide, and it was not until methyl bromide was phased out 30 years later (being a major ozone-depleting substance) (Ristaino and Thomas, 1997), that Taylor’s new nematicide era finally began to emerge. In response to the phasing out of methyl bromide, many companies realized the new opportunity at hand, and initiated new nematicide discovery programs.

## How new nematicides are being discovered?

Ideas for new nematicides can come from a variety of sources, such as chemical libraries, scientific literature, natural products, and patents. Pharmaceutical and crop protection companies may have libraries of several million compounds, and often have exchange agreements. Cross-industry patent searches, especially of newly released patents, are another common source for starting points. Historically, the ‘spray and pray’ approach, meaning a large number of unknown or novel compounds are evaluated in some type of high-throughput screening against a certain target pest, and visually evaluated for efficacy, has been the most commonly used approach (Drews et al., 2012; Slomczynska et al., 2015). If a new molecule shows activity, extensive chemical research is then used to identify and modify its structure to improve its performance. While this approach is still common, random screening of compounds has given way to more targeted efforts, such as combinatorial chemistry (Lindell et al., 2009) and structure-based design (Baker and Umetsu, 2001). The idea is that compounds fit some a priori hypothesis (Lamberth et al., 2013) or are pre-filtered for agrochemical-like properties (Jeschke, 2016). The rapid advances in the ‘omics’ fields have created more opportunities for target-based discovery and the synthesis of specific molecules that bind to specific proteins; however, this approach has not yet lived up to the high expectations.

In the end, the best place to find biological activity is still in other biologically active compounds, such as existing pesticides, pharmaceuticals, and natural products. With the demand for biological products in agriculture growing steadily, many companies now have microbial and natural product libraries. One reason for the suitability of natural products in agriculture, or at least as lead candidates for further discovery, is their biologically relevant chemical diversity. Natural products have evolved with and against their biological targets, which is often manifested in high affinity interactions. Throughout history, nature has been a prolific source of drugs (Dias et al., 2012), as well as of some highly effective pesticides. There are many examples of pesticides that are natural products or derivatives thereof, such as avermectins (insecticide/nematicide), spinosyns (insecticide), pyrethrins (insecticide), strobilurins (fungicide), and triketones (herbicide) (Cantrell et al., 2012). Between 1979 and 2010, natural products accounted for almost 70% of all new active ingredient registrations (Cantrell et al., 2012). Biological nematicides will likely become more important in the future. They will not be covered in this review but merit another full review of their own.

## How to detect nematicidal activity – what assay to use?

Sometimes new active compounds are discovered accidentally, such as with penicillin (Fleming, 1929), but in most cases, they are the result of a targeted discovery effort, using one or more specific assays. A nematicide discovery program is only as good as the biological assay that is in place to detect its activity, and the ‘best’ screening method is always a compromise between speed and accuracy. This will primarily depend on the target and the throughput. Different systems and nematode models can be used, including in vitro or whole plant assays. Nematicidal activity can be measured by visual observations of the body shape or movement of nematodes. However, such observations are not always the most reliable, as nematodes may respond very differently depending on the mode-of-action of the tested compound. Another concern is the timing of the evaluation or observation. If the observation is done too soon, slower-acting compounds may be missed, if done too late, nematodes may be able to recover. Ultimately, the time it takes for mass screening of large numbers of compounds is critical, and a balance needs to be found between accuracy and efficiency. While it is impossible to exclude errors, they need to be minimized. It is especially critical to minimize type II errors or false negatives (no activity is found where there is activity), as this may lead to missing out on potentially promising compounds.

The bacterial-feeding nematode *C*. *elegans* is frequently used as a model to find new nematicides as it can be easily used in high-throughput screening (Slomczynska et al., 2015). However, the nematode is not a plant parasite, which means that its suppression might not translate well to plant-parasitic nematodes. For example, a new nematicide, fluazaindolizine, has no activity against *C. elegans*, but good activity against plant-parasitic nematodes (Lahm et al., 2017). Most of the current nematicide discovery screens use root-knot nematode (*Meloidogyne* spp.) as a model. Root-knot nematodes are easy to culture, they cause visible and quantifiable root symptoms, and they are the most important plant-parasitic nematodes worldwide. Another consideration is whether to use in vitro assays (evaluating nematode movement) or plant-based assays (evaluating root infection). Plant-based assays are usually more time consuming, but also more realistic, and more likely to cover a broader range of possible mode-of-actions.

## From discovery to product – what does it take?

**Figure 2:**
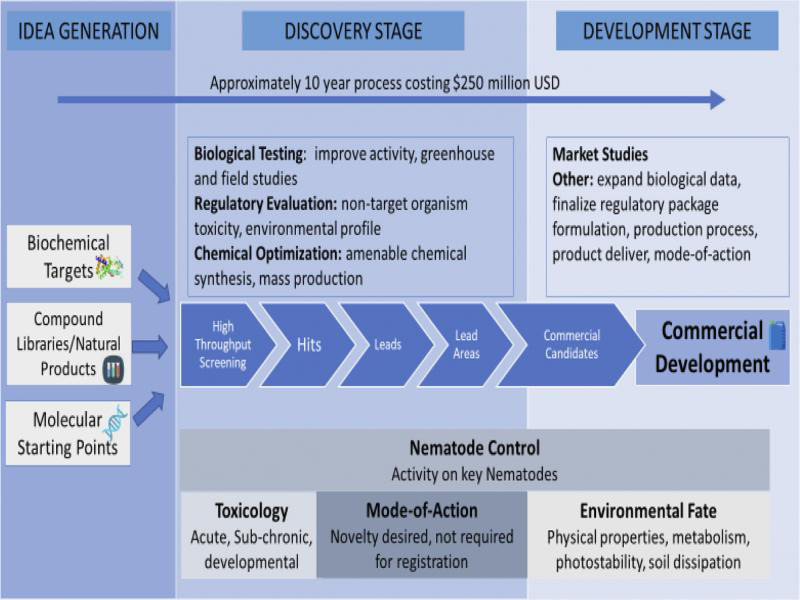
Typical process for discovery and development of new nematicides.

Once the initial nematicidal activity has been found, the focus is to improve this activity. At the same time, other activities need to be done, such as studies on toxicology and environmental impact, mode-of-action, soil behavior, formulations, patent situation, cost of manufacturing, and use rates and potential uses. The early discovery research is always confidential, and it is typically not until a few years before registration, that the research is made public. Figure 2 outlines a nematicide discovery process in industry, starting from idea generation, to the discovery process, and up to the commercial development phase. The entire process is highly integrated and requires a wide range of experts working together. It is estimated that only one in 140,000 active ingredients discovered today will pass the rigorous testing requirements to become a registered pest management product (Sparks, 2013).

Majority of research done on new nematicides is focused on their risk assessment on the environment and human health, that include potential impacts on wildlife, fish, plants, and other non-target organisms. Safety to non-target organisms is becoming increasingly important, and safety studies include organisms such as collembola, soil and predatory mites, honeybees, spiders, and water fleas. For soil-applied nematicides, the product’s impact on the soil environment is an important source of concern, and many regulatory requirements are put in place to address this. Pesticide registration nowadays is a very complex, highly regulated, and involved process from start to finish.

The cost of bringing a new chemical active ingredient to market is increasing every year, and is now estimated to be more than US$250 million, about 10-fold what it was in the 1960s (Sparks, 2013). Similarly, the average time from product discovery to market launch has increased and is now >10 years. This trend will probably continue, making it increasingly harder for smaller firms to bring new products to the market, as they simply cannot afford to invest the time and money, much less deal with the substantial amount of regulatory documentation.

## Characteristics and mode-of-action of new generation of nematicides

The new nematicides that will be discussed are listed in Table 2. These new nematicides are very different from previous products, in large part due to the regulatory requirements on human and environmental safety. Soil behavior – such as leaching potential, soil persistence, effects on beneficial soil organisms, degradation and metabolism pathways – is now a critical component of the registration and development process (Table 3). Ideally, a nematicide will only affect plant-parasitic nematodes, work consistently, does not leave residue in the soil or plants, is easy and safe to apply, and is inexpensive. Combining all these traits is a challenge, but the pay-off could be quite substantial. The new generation of nematicides certainly have a much better profile in terms of operator safety and selectivity, and with more companies stepping up their efforts, more nematicides will continue to become available in coming years. An overview of the most significant new chemical nematicides to emerge in the last decade is provided (Table 2).

**Table 2.** Characteristics of new synthetic nematicides as compared to older products (fumigant and carbamate nematicides).

Chemical name	Chemical structure	Water solubility	Soil 1/2 life	Mode-of-action	Signal words
Fumigant (1,3-D)	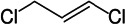	Gas	Short < 14 d	unknown	Danger
Carbamate (oxamyl)	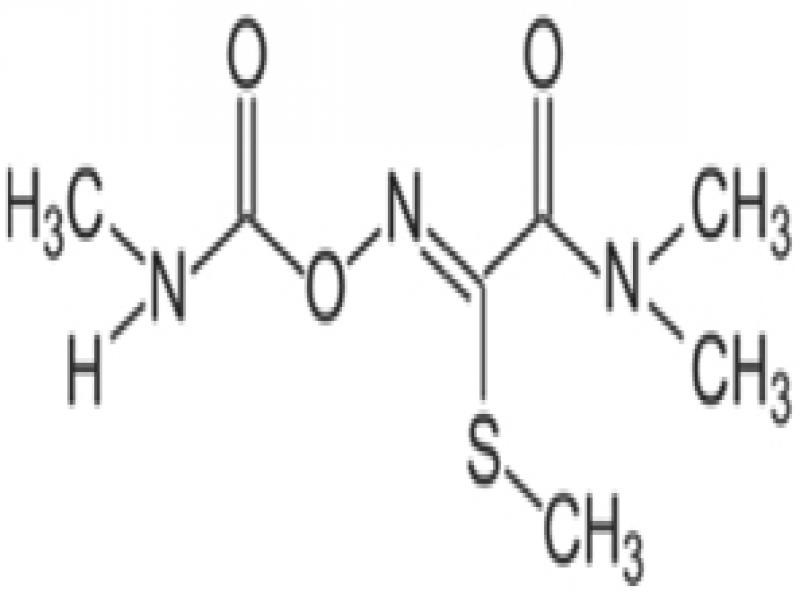	240,000 ppm	Short 7 d	AChE^a^	Danger
Fluensulfone	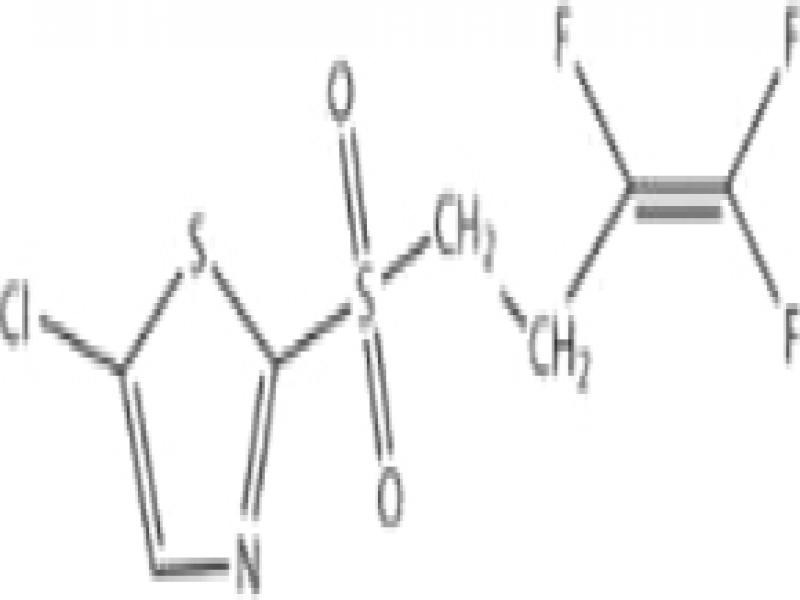	545 ppm	Short 7-17 d	Beta oxidation inhibitor	Caution
Fluopyram	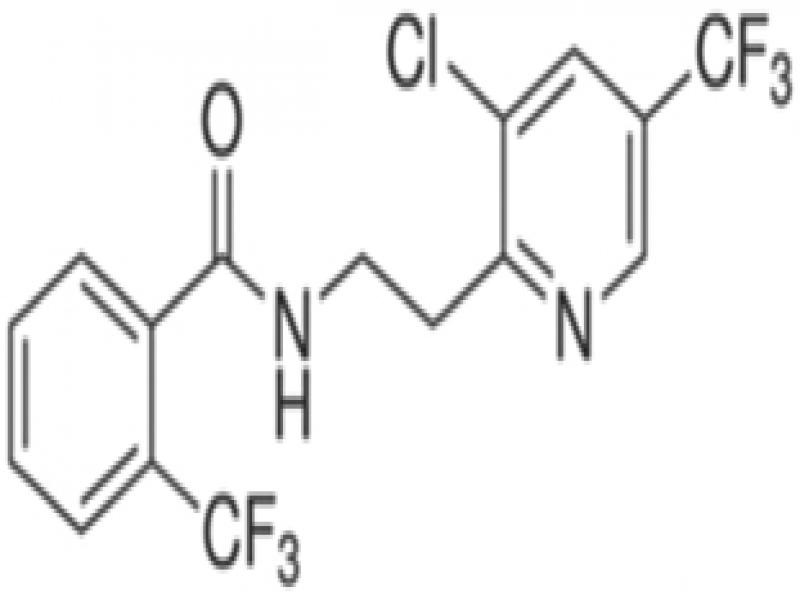	10 ppm	Long > 200 d	SDHI^b^	Caution
Fluazaindolizine	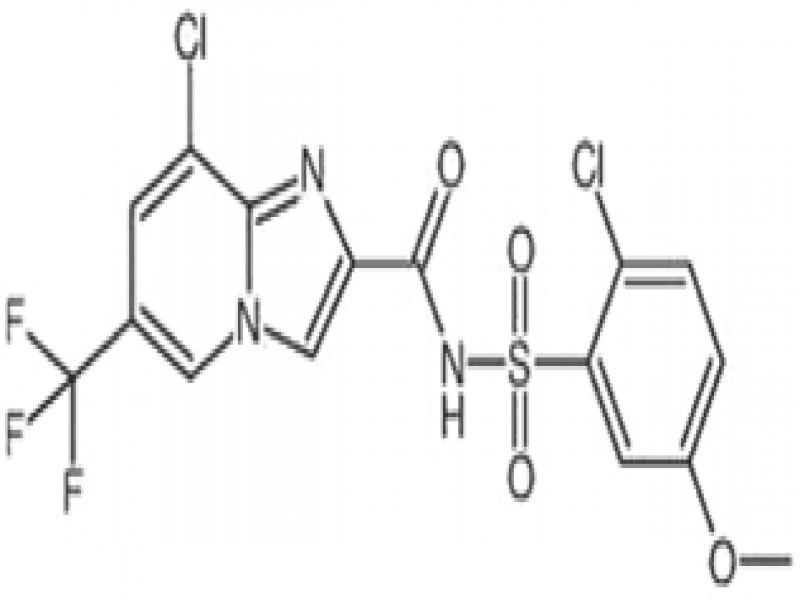	2000 ppm	Medium 30 d	unknown	TBD
Spirotetramat	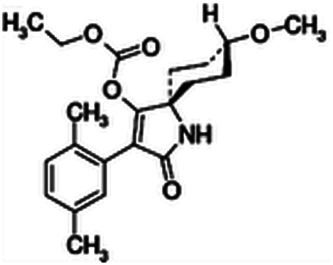	30 ppm	Short (< 1 d)	ACC^c^ inhibitor	Warning
Tioxazafen	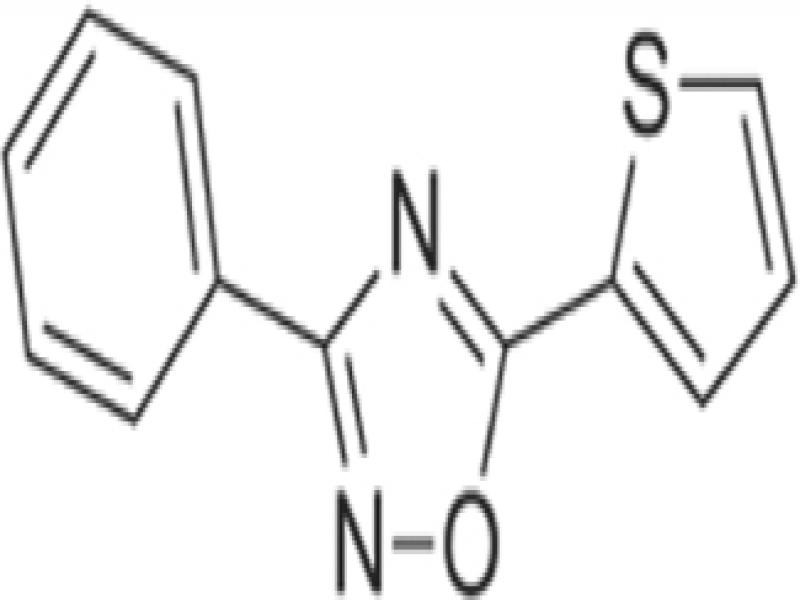	1.24 PPM	Long (48-303 d)	Disrupts ribosomal activity	Caution

**Notes:** ^a^AChE = acetyl cholinesterase inhibition; ^b^SDHI = succinate dehydrogenase inhibition; ^c^ACC = Acetyl-CoA carboxylase.

**Table 3.** Characteristics of the ideal nematicide.

	Grower perspective	Regulator perspective
Intrinsic activity	Broad-spectrum, controls all parasitic nematodes	Selectivity (safe to non-target/beneficial organisms)
Soil behavior	Good soil movement and long soil residual activity	No leaching and low soil persistence
Plant behavior	Systemic activity, low phytotoxicity	No crop residues, no negative impact on produce quality
Application	Flexibility, low rates	Safe to handlers, low human toxicity

*Tioxazafen* (Nemastrike^®^, Monsanto/Bayer) was originally discovered by Divergence, which was acquired by Monsanto in 2011, which in turn was acquired by Bayer in 2018 (Table 3). The compound is a new systemic nematicide belonging to the chemical class oxadiazoles (Slomczynska et al., 2015). Its mode-of-action may be linked to disruption of ribosomal activity of nematodes. Few data have been made available on this compound, which is registered as a seed treatment for corn, cotton or soybean only. At the time of writing, tioxazafen was no longer offered for use in 2020, citing safety concerns.

*Spirotetramat* (Movento^®^, Monsanto/Bayer) is a spirocyclic tetramic acid derivative and was first marketed as a fully systemic insecticide (Nauen et al., 2008; Table 2). Spirotetramat has unique translocation properties in that it is translocated within the entire vascular system (upwards and downwards through the xylem and phloem, respectively). To be active against nematodes, the compound needs to be hydrolyzed in the plant to the active spirotetramat-enol form (Vang et al., 2016). In *C. elegans*, spirotetramat-enol leads to arrested larval development and disruption of the life cycle (Vang et al., 2016; Gutbrod et al., 2018). The nematicide also inhibits acetyl-CoA carboxylase activity, storage of lipids, fatty acid composition, and disruption of surface coat synthesis in *C. elegans* (Gutbrod et al., 2018). Silencing of acetyl-CoA carboxylase in *Heterodera schachtii* by RNAi mimicked the effects of spitrotetramat-enol, indicating that the mode-of-action is inhibition of acetyl-CoA carboxylase (Gutbrod et al., 2018). Concentrations of spirotetramat-enol necessary to result in 95% of a *C. elegans* population having arrested development were 44 to 48 ppm (Vang et al., 2016). There is some evidence that spirotetramat-enol needs to be ingested by plant-parasitic nematodes to elicit an effect. *Meloidogyne incognita* and *Mesocriconema xenoplax* were exposed to 0.017 and 0.026 kg/ha spirotetramat in vitro, which reduced mobility of *M. xenoplax* but not of *M. incognita* (Shirley et al., 2019). In another study, egg hatch of *Rotylenchuls reniformis* was not inhibited after exposure to spirotetramat (Waisen et al., 2019).

Fluensulfone, fluopyram, and fluazindolizine are new nematicides that all have a trifluoro (3-F) group in their molecular structures and are hereby referred to as 3-F nematicides. These nematicides have a much safer toxicity profile than the older nematicides (fumigants, organophosphates, carbamates) (Table 2). However, they are quite different in terms of their chemical and physical properties, and their modes-of-action.

*Fluensulfone* (Nimitz^®^, Adama) was the first of the new chemical nematicides and was first registered in the US in 2014 (EPA, 2014). Fluensulfone is a member of the class of 1,3-thiazoles and was originally discovered by Nihon Bayer in 2001. This compound is a specific nematicide and was the first of its kind to become registered. Fluensulfone has a soil half-life up to 36 days (Ludlow, 2015b, Table 2). Under field conditions, fluensulfone had a 50% dissipation rate (DT_50_) between 23.7 and 24.3 days depending on soil type, similar to that of fosthiazate (Norshie et al., 2017). When treated with similar doses of fluensulfone*, Caenorhabditis elegans* and *M. incognita* respond with immobility and eventual death (Kearn et al., 2014). This included *C. elegans* mutants resistant to organophosphates and carbamates, indicating that cholinesterase inhibition is not the target for this compound. The unpublished mode-of-action of fluensulfone is that it is a fatty acid beta oxidation inhibitor. More recently, Kearn et al. (2017) have found that in *G. pallida* second-stage juveniles (J2) exposed to fluensulfone exposure led to increased lipid content, loss of cell viability, and tissue degeneration symptoms not seen in adult and dauer *C. elegans* (Kearn et al., 2017). Additionally, exposed *Globodera pallida* J2 had reduced stylet thrusting and reduced mobility along with coiling posture; the rate of these symptoms correlated strongly with the concentration of fluensulfone. The higher the dosage the faster symptoms appeared; at 146 ppm of fluensulfone *G. pallida* J2 experienced the coiling posture after just 30 min.

Several *in vitro* studies have been conducted to determine the effects of fluensulfone on *Meloidogyne* species. Doses as low as 25 ppm impacted *M. incognita* J2 activity after 24 hr exposure, and egg hatch was reduced at 95 ppm (Moreira and Desaeger, 2019; Wram and Zasada, 2019). *Meloidogyne* species and populations within a species varied in their response to fluensulfone (Oka and Saroya, 2019). *Meloidogyne incognita* was more sensitive to fluensulfone than *M. javanica*. The fluensulfone 17 hr LC_50_ was ten-fold (0.48 vs 0.12 mg/L) different between two *M. incognita* populations. Exposing *M. javanica* J2 to fluensulfone at sublethal concentrations for 17 hr was able to reduce the number of J2 attracted to lettuce root tips in pluronic agar and those that invaded produced smaller galls (Oka and Saroya, 2019). Migratory plant-parasitic nematodes showed different responses *in vitro* (Oka, 2014). *Bursaphelenchus xylophilus* and *Ditylenchus dipsaci* were not impacted by exposure to fluensulfone at high concentrations (16 mg/L) after 48 hr, unlike *Aphelenchoides besseyi* and *Aphelenchoides fragariae* which had > 50% immobility after 48 hr of exposure. More than 60% of *Pratylenchus penetrans* and *P. thornei* were immobilized after exposure to fluensulfone of 4 mg/L and stayed immobilized even after removal of the compound (Oka, 2014). *Xiphinema index* was also impacted irreversibly by exposure to fluensulfone at doses as low as 1 mg/L with 60% immobility after 48 hr of exposure and a 24 hr rinse (Oka, 2014).

*Fluopyram* (Verango^®^, Velum^®^, Indemnify®, Bayer CropScience) was initially discovered and patented by Bayer as a fungicide (WIPO, 2005), and was first registered as a fungicide in 2012 under the trade name Luna (EPA, 2012). The nematicidal activity of fluopyram was not known or disclosed until 2008, when Nihon Nohyaku CO filed a nematicide use patent for fluopyram. The first nematicide registration was in Honduras (Agrow, 2013) on banana under the name Verango, and later in South Africa (AgroNews, 2015) and US (EPA, 2015) under the name Velum. Fluopyram is a member of the class of benzamides, and a Fungicide Resistance Action Committee code 7 fungicide. It is an inhibitor of the enzyme succinate dehydrogenase in fungi (Veloukas and Karaoglanidis, 2012). Heiken (2017) confirmed this to be the likely mode-of-action in nematodes, demonstrating that succinate dehydrogenase knockdown mutants of *C. elegans* had a roughly 2.6-fold increase in sensitivity to fluopyram. Unlike the other 3-F nematicides, fluopyram has a very long soil half-life, up to 746 days depending on soil type (Ludlow 2015a, Table 2).

Fluopyram is a very fast-acting and potent nematicide, with *M. incognita* J2 exposed to fluopyram at 5.58 ppm for 2 hr showing reduced mobility by 80%, and the 24 hr ED_50_ for *M. incogntia* being as low as 1 ppm (Faske and Hurd, 2015; Wram and Zasada, 2019). Fluopyram also showed good activity against *Rotylenchulus reniformis*, with the 2-hr EC_50_ roughly 2x that for *M. incognita* (Faske and Hurd, 2015). However, fluopyram also showed to be nematistatic in *in vitro* assays, with a 58% recovery of mobility of *M. incognita* J2 after removal of the nematicide. Although nematistatic, in that same study, exposure to fluopyram for 1 hr to 1.3 and 3.3 ug/ml for *M. incognita* and *R. reniformis* significantly reduced reproduction of nematodes on tomato. Wram and Zasada (2019) also demonstrated that fluopyram is a poor ovicide, with only a slight reduction in *M. incognita* egg hatch at 2.5 ppm.

*Fluazaindolizine* (Salibro^®^, DuPont/Corteva) is the latest of the new chemical nematicides and is expected to be registered in 2021 (Lahm et al., 2017). Like fluensulfone, fluazaindolizine specifically targets nematodes, and no other (fungicidal or insecticidal) activity has been reported. Fluazaindolizine is a member of the class of carboxamides. Its mode-of-action is unknown, however it is distinct from that of carbamates/organophosphates, or any other known nematicides (Lahm et al., 2017). Fluazaindolizine was unable to inhibit motility and mortality of *C. elegans* adults and *Drosophila melanogaster* egg and adult stages at concentrations of 200 and 300 ppm, respectively (Lahm et al., 2017). However, *M. incognita* J2 exposed to 5 to 50 ppm of fluazaindolizine were increasingly immobile and eventually dead 24 to 96 hr after exposure (Lahm et al., 2017).

Fluazaindolizine has irreversible effects on *M. incognita* even after 24 hr of exposure (Thoden and Wiles, 2019; Wram and Zasada, 2019). However, based on nematode motility, a 24-hr EC_50_ for *M. incognita* J2 for this compound is over 2× that of fluensulfone and over 200× that of fluopyram (Wram and Zasada, 2019), making this a slower-acting nematicide. In a soil environment, exposure to fluazaindolizine reduced the ability of *M. hapla* and *M. incognita* to move through sand at concentrations of 50 ppm (Thoden and Wiles, 2019). Fluazaindolizine was also a poor ovicide, with reductions in *M. incognita* egg hatch only at concentrations over 250 ppm for 7 days of exposure (Thoden and Wiles, 2019; Wram and Zasada, 2019). When effects of this compound on free-living nematodes were considered, there was no reduction in *Acrobeloides buetschlii* egg hatch over 5 days with exposure to fluazaindolizine at concentrations as high as 250 ppm (Thoden and Wiles, 2019). There was also no effect on *A. buetschlii* motility at concentrations as high as 250 ppm after 144 hr of exposure.

Similar to fluensulfone, there can be some variation in how populations of the same plant-parasitic nematode species respond to fluazaindolizine. Five populations of *M. incognita* and five populations of *M. javanica* were examined after exposure to fluazaindolizine for their effects on J2 mobility and motility (Thoden et al., 2019). After 24 hr of exposure to fluazaindolizine at 50 ppm the percentage of affected *Meloidogyne* J2 in all populations tested ranged from 42 to 86%. When *Meloidogyne* J2 were pre-exposed for 24 hr at 50 ppm, the ability to move through a sand layer varied from 53 to 100% across populations. In general, *M. javanica* mobility and motility was slightly less impacted by exposure to fluazaindolizine compared with *M. incognita*.

## Efficacy of new chemical nematicides in greenhouse and field experiments

Application rates for the new nematicides are similar or somewhat lower than rates of old organophosphate or carbamate nematicides (1-2 kg ai/ha), and much lower compared to fumigant application rates of 200 to 300 kg ai/ha. Rates of application for the new nematicides range from 1 to 2 kg ai/ha for fluensulfone and fluazaindolizine, and less than 0.5 to 0.7 kg ai/ha for fluopyram and spirotetramat.

While initially marketed as an insecticide, spirotetramat began to receive attention as a nematicide in 2009 (McKenry et al., 2009) (Table 4). Since this initial report, there have been several greenhouse and field studies evaluating the nematicide against a range of plant-parasitic nematodes; in all cases spirotetramat was applied foliarly. Optimal efficacy occurred when spirotetramat application coincided with early stages of nematode root infection (Vang et al., 2016). Single (0.017 kg/ha) and dual applications (0.017 and 0.26 kg/ha) of spirotetramat to peach in a greenhouse study reduced *M. incognita* densities by at least 54% compared to untreated controls (Shirley et al., 2019). In the same study, no effects were observed on final population densities of *M. xenoplax* on peach with the same treatments. Other greenhouse studies with spirotetramat did not demonstrate any measurable effects on *P. penetrans* (Zasada et al., 2010), *M. incognita* (Baidoo et al., 2017), and *Aphelenchoides ritzemabosi* (Chalanska et al., 2017). In a number of tree and fruit crop trials in California with spirotetramat, percentage reductions of plant-parasitic nematodes (*Xiphinema americanum, X. index, Pratylenchus vulnus, Tylenchulus semipenetrans, Meloidogyne* spp., and *Criconemoides xenoplax*) were around 50% across the different crops, provided that irrigation was delayed for two weeks following treatment (McKenry et al., 2009). In the Pacific Northwest (Smiley et al., 2011, 2012), spirotetramat suppressed population densities of *H. avenae* but not of *P. neglectus*. It was also found that application timing was important, with greater efficacy against *H. avenae* when the product was applied before white females became apparent on roots. Spirotetramat did not suppress *M. incognita* in lima bean (Jones et al., 2017).

**Table 4.** Summary of the literature evaluating new reduced-risk agricultural nematicides.

		Experimental venue			
Nematicide	Nematode	Laboratory	Greenhouse	Field	Reference
Spirotetramat	*Aphelenchoides ritzemabosi*		Nursery plant		Chalanska et al. (2017)
	*Heterodera avenae*			Wheat	Smiley et al. (2011, 2012)
	*Meloidogyne incognita*		Lima bean	Lima bean	Jones et al. (2017)
	*M. incognita*	X	Peach		Shirley et al. (2019)
	*M. incognita*		Nursery plant	Nursery plant	Baidoo et al. (2017)
	*Meloidogyne* spp.			Multiple perennials	McKenry et al. (2009)
	*Mesocriconema xenoplax*	X	Peach		Shirley et al. (2019)
	*M. xenoplax*			Multiple perennials	McKenry et al. (2009)
	*Pratylenchus neglectus*			Wheat	Smiley et al. (2012)
	*Pratylenchus penetrans*		Raspberry		Zasada et al. (2010)
	*Pratylenchus vulnus*			Multiple perennials	McKenry et al. (2011)
	*Rotylenchulus reniformis*	X			Waisen (2015)
	*Tylenchulus semipenetrans*			Multiple perennials	McKenry et al. (2009)
	*Xiphinema americanum*			Multiple perennials	McKenry et al. (2009)
	*Xiphinema index*			Multiple perennials	McKenry et al. (2009)
Fluopyram	*Belonolaimus longicaudatus*			Strawberry	Watson and Desaeger (2019)
	*Heterodera glycines*	X	Soybean		Heiken (2017)
	*Meloidogyne hapla*			Strawberry	Watson and Desaeger (2019)
	*M. incognita*	X	Tomato		Wram and Zasada (2019)
	*M. incognita*	X	Tomato		Faske and Hurd (2015)
	*M. incognita*	X	Tomato		Heiken (2017)
	*M. incognita*		Lima bean	Lima bean	Jones et al. (2017)
	*M. incognita*			Cucumber	Hajihassani et al. (2019)
	*M. incognita*			Carrot	Becker et al. (2019)
	*M. incognita*		Tomato		Silva et al. (2019)
	*Meloidogyne javanica*			Tomato	Desaeger and Watson (2019)
	*P. penetrans*			Strawberry	Watson and Desaeger (2019)
	*R. reniformis*	x	Tomato		Faske and Hurd (2016)
Fluensulfone	*B. longicaudatus*			Strawberry	Watson and Desaeger (2019)
	*B. longicaudatus*			Potato	Grabau et al. (2019)
	*Globodera pallida*			Potato	Norshie et al. (2016)
	*Longidorus vineacola*			Pepper	Oka (2019)
	*M. hapla*			Strawberry	Watson and Desaeger (2019)
	*M. incognita*	x	Tomato		Wram and Zasada (2019)
	*M. incognita*			Carrot	Becker et al. (2019)
	*M. incognita*		Tomato		Silva et al. (2019)
	*M. incognita*		Lima bean	Lima bean	Jones et al. (2017)
	*M. incognita*			Cucumber	Hajihassani et al. (2019)
	*M. incognita*			Sweet Potato	Ploeg et al. (2019)
	*M. incognita*			Cucumber	Morris et al. (2016)
	*M. javanica*			Tomato	Desaeger and Watson (2019)
	*Paratrichodorus* sp.			Potato	Grabau et al. (2019)
	*P. penetrans*			Lettuce	Oka (2019)
	*P. penetrans*			Strawberry	Watson and Desaeger (2019)
	*P. thornei*			Chickpea	Oka (2019)
	*Pratylenchus* sp.			Potato	Grabau et al. (2019)
	*X. index*			Fig	Oka (2019)
Fluazaindolizine	*B. longicaudatus*			Strawberry	Watson and Desaeger (2019)
	*M. hapla*			Strawberry	Watson and Desaeger (2019)
	*M. incognita*	x	Tomato		Wram and Zasada (2019)
	*M. incognita*		Tomato		Silva et al. (2019)
	*M. incognita*			Carrot	Becker et al. (2019)
	*M. incognita*	X			Thoden and Wiles (2019)
	*M. incognita*			Cucumber	Hajihassani et al. (2019)
	*M. javanica*			Tomato	Desaeger and Watson (2019)
	*P. penetrans*			Strawberry	Watson and Desaeger (2019)

**Note:** Empty fields within an experimental venue indicates that there is no data available.

The 3-F nematicides (fluensulfone, fluopyram, fluazaindolizine) have been evaluated on a variety of crops for their ability to suppress a diversity of plant-parasitic nematodes (Table 4). However, most have focused on their efficacy against *Meloidogyne* species in vegetable crops. Several greenhouse studies evaluated the efficacy of these compounds on controlling *M. incognita* on susceptible tomato cultivars at concentrations ranging from labeled rates to 24-hr sublethal pre-exposure doses. At sublethal doses fluazaindolizine and fluensulfone suppressed *M. incognita* reproduction more than fluopyram, although actual sublethal doses were much lower for the latter (Thoden and Wiles, 2019; Wram and Zasada, 2019). In another study, half and full labeled rates of any of these compounds did not suppress reproduction of *M. incognita* (Silva et al., 2019).

As stated above, it appears that fluopyram may be a poor ovicide. Fluopyram applied at the labeled rate (249 g a.i./ha) to the soil surface of tomato plants two days post inoculation with *M. incognita* eggs had no impact on nematode reproduction when compared to the untreated control (Heiken, 2017). However, fluopyram applied at the labeled rate two weeks after tomatoes were inoculated with *M. incognita* eggs, reduced the number of eggs per gram of root by 91% compared with the untreated control, demonstrating that application timing is important.

Both fluopyram and fluensulfone were effective nematicides against *M. incognita* in lima bean in both greenhouse and microplot studies (Jones et al., 2017). With similar control to oxamyl, fluopyram at a rate of 0.22 L a.i./ha reduced galling 55 and 64%. Fluensulfone was the most effective against *M. incognita* in this study, with > 81% reduction in galling on lima beans when applied at 2.34 L a.i./ha.

The impact of initial nematode density on efficacy of the 3-F compounds has also been explored (Hajihassani et al., 2019). In this microplot study, initial population densities of *M. incognita* ranged from 1,000 to 20,000 J2/microplot. Fluensulfone and fluazaindolizine had the lowest final soil population levels compared with oxamyl and fluopyram. At low inoculation densities there was no difference between the nematicide in reducing root galls, but at high nematode densities fluopyram, fluazaindolizine, and fluensulfone had greater gall reduction compared to oxamyl.

Becker et al. (2019) found contradictory results when examining the effects of these compounds on carrot production over multiple years. Fluensulfone and fluopyram were unable to consistently lower final population densities of *M. incognita*, unlike fluazaindolizine. Fluopyram was also less consistent in reducing harvest galling and only protected the top 1/3 of the carrot tap root, unlike fluensulfone and fluazaindolizine. In cucumber, drip applied fluensulfone at a rate of 3.0 kg a.i./ha was able to reduce *M. incognita* J2 population densities by 73% along with a reduction in root galling (Morris et al., 2016).

Desaeger and Watson (2019) also found that the use of these compounds could help prevent rapid re-infestation of roots. When field grown tomatoes were treated with drip applied non-fumigant nematicides, fluensulfone had the most consistent suppression of *M. javanica* compared to treatments of fluazaindolizine, fluopyram, and treatment combinations of oxamyl and fluopyram, oxamyl and fluazaindolizine, and fluensulfone and oxamyl. In chloropicrin treated soils there was an increase in nematode population at the end of the growing season, unlike in non-fumigant nematicide treated soils that had consistently lower levels of *M. javanica*.

When evaluating these compounds for control of nematodes outside of *Meloidogyne* species, there have been mixed results. Watson and Desaeger (2019) conducted a field study with *Belonolaimus longicaudatus, Meloidogyne hapla, Pratylenchus penetrans* and found that of the non-fumigant nematicides tested (fluopyram, fluensulfone, fluazaindolizine) fluopyram was the only nematicide to reduce *B. longicaudatus* and improve strawberry yield. Fluopyram was able to somewhat reduce *P. penetrans* in soil and roots compared with the control and metam-potassium, but this effect was not significant. Neither fluensulfone or fluazaindolizine were able to suppress *B. longicaudatus, M. hapla, P. penetrans* or increase vigor or yield of strawberry.

In a greenhouse study, Oka (2019) also evaluated the effects of fluensulfone exposure pre- and post-plant on *Xiphinema index* and *Longidorus vineacola* in fig and pepper. When soil infested with *X. index* was treated with 5 mg/L of fluensulfone 1 week prior to introducing a fig transplant, it reduced the number of *X. index* recovered with no difference observed with rates of fluensulfone up to 20 mg/L. *Longidorus vineacola* was more sensitive to fluensulfone, with 2 mg/L fluensulfone applied one-week prior to transplant showing to suppress this nematode (Oka, 2019). Treatments that were applied one-week post-plant were less effective for both nematodes with >2× pre-plant effective doses needed to reduce the number of *X. index* and *L. vineacola* recovered (Oka, 2019). In a greenhouse assay, fluensulfone applied at 2 mg/L reduced population densities of *P. penetrans* and *P. thornei* growing on lettuce and chickpea, respectively (Oka, 2014).

Several studies have evaluated the effects of fluensulfone on plant-parasitic nematodes infecting tuber crops like potato and sweet potato (Table 4). A full rate of fluensulfone in liquid and granular form resulted in comparable suppression of *G. pallida* to that by fosthiazate, but was not as effective as oxamyl (Norshie et al., 2016). Fluensulfone suppressed *B. longicaudatus*, *Pratylenchus* sp. and *Paratrichodorus* sp. on potato over three years (Grabau et al., 2019). Fluensuflone at all rates tested (2.92, 4.11, 5.87, 8.20 L/ha) consistently reduced densities of *B. longicaudatus* at harvest and was comparable to treatment with Telone II (1,3-dichloropropene at 61 L/ha). However, fluensulfone did not consistently suppress *Pratylenchus* sp. or *Paratrichodorus* sp. across years. In the same study (Grabau et al., 2019), potato yield was consistently higher in fluensulfone treated plots, especially with lower rates of fluensulfone. However, both Telone II and fluensulfone resulted in end of harvest final nematode population densities that were greater than initial densities of nematodes. Ploeg et al. (2019) explored the effects of fluensulfone and application timing of fluensulfone on suppression of *M. incognita* on sweet potato. Fluensulfone was applied at 3.36 kg/ha 2 to 7 days pre-plant, followed by 2 post-plant applications of 1.68 kg/ha at 26 and 58 days. Marketable yield was increased for both treatments of fluensulfone, but similarly to results from potato (Grabau et al., 2019), final soil population densities of *M. incognita* were 8 to 13 times higher than initial population densities. However, Ploeg et al. (2019) found that both fluensulfone treatments were able to reduce *M. incognita* eggs/g of sweet potato root by 80%, which could contribute to the increased marketable yield.

As indicated, the bulk of efficacy data of 3-F nematicides is on *Meloidogyne* spp., and all products seem to be quite effective against these nematodes (Oka et al., 2012; Morris et al., 2016; Heiken, 2017; Jones et al., 2017; Desaeger and Watson, 2019) (Table 4). Much less data is available on other plant-parasitic nematodes, but as stated above, indications are that several nematodes such as *Pratylenchus* spp. and *Belonolaimus longicaudatus* may be more tolerant to fluazaindolizine, and a lesser extent fluensulfone (Grabau et al., 2019; Watson and Desaeger, 2019). Although fluopyram overall shows more broad-spectrum activity (Moreira and Desaeger, 2019), it does not seem to affect lance nematodes (*Hoplolaimus* spp.) in turf (Crow, 2017). It is not clear why certain plant-parasitic nematodes might be less sensitive, but possibly differences in cuticle permeability, or detoxification mechanisms inside the nematode are involved. This may also explain their reported relative inactivity against non-plant-parasitic nematodes, including entomopathogenic nematodes (Moreira and Desaeger, 2019; Thoden and Wiles, 2019). Fluopyram, as stated earlier, seems to have more broad-spectrum activity, including against non-plant-parasitic nematodes (Moreira and Desaeger, 2019; Waldo et al., 2019). More long-term field studies are needed to verify this, but the relative inactivity towards beneficial nematodes would certainly be a good thing.

Differences in nematicide efficacy across trials may be due to the physical properties of the new products, especially their solubility in water and half-life in soil (Table 3). Water solubility is important in that it will determine how well the molecule moves through the soil profile. Higher water solubility will give better soil coverage and distribution of the active but will also increase the risk of leaching. Soil half-life determines how long the molecule stays active in the soil. Longer soil half-life will give longer soil residual activity and more extended nematode control, but also increases the risk of soil accumulation. Fluensulfone and fluazaindolizine are relatively similar in terms of solubility and soil half-life, but fluopyram is quite different, having low water solubility, and a much longer soil half-life (Table 3). Overall, all the 3-F nematicides have much lower water solubility, but longer soil half-lives than oxamyl. A lack of uniform soil distribution, combined with the typically patchy field distribution of nematodes, could explain some of the variability observed in the above field trials. Also, the lack of standardization in terms of sampling procedure, sampling time, and extraction method is another source of variability. Like in any other discipline, a keen understanding of the pest, i.e. nematode biology and plant-nematode interactions, is critical to understand, interpret, and validate the inherent variability of nematode field trial data. It should come as no surprise that a background specific to applied nematology is now a discipline in high demand, but also in short supply. Fortunately, both industry and universities have started to notice this gap in expertise, and many have started hiring applied nematologists again.

## Future prospect and research needed for new generation of nematicides

The practice of soil fumigation is facing increasing societal and regulatory pressure, but nevertheless remains the primary nematode management tool in the production of many high-value crops. Soil fumigation is convenient, as it gives growers weed, soil disease, and nematode control all at once. Also, fumigants are often the only nematicides available to growers, with many crops not having a single registered nematicide available until recently. The recent entry of safer and more selective nematicide alternatives is welcome news for growers, but questions remain about their efficacy and adoptability. Their selectivity, both among plant-parasitic and non-parasitic nematodes, needs to be studied further, as well as how these new products can best be integrated into existing nematode management programs.

Nematode resistance was never a major concern for nematicides in the past, probably due to the broad-spectrum nature, and relatively limited use of most of the old products. With the new nematicides being more selective, and potentially used more frequently, resistance may be more likely to occur. For instance, SDHI compounds like fluopyram, having long soil persistence and similar mode-of-action towards fungi and nematodes, are likely to put significant selection pressure on target nematodes. It is also well-known that many of the older organophosphate and carbamate nematicides can lose efficacy over time due to accelerated degradation in the soil caused by microbial adaptation (Smelt et al., 1987; Johnson, 1998). Certainly, this is something that should be monitored for all the new 3-F nematicides as well. Recently, a new IRAC (Insecticide Resistance Action Committee) Nematode Working Group was established to investigate the resistance risk of new nematicides and to develop a mode-of-action classification scheme similar to insecticides and acaricides (IRAC, 2019).

The future impact of the new nematicides will depend on (i) how effective they prove to be under field conditions – they have to show they can reduce nematode damage consistently, and improve crop vigor and yield, (ii) the future regulatory status of fumigants – if regulatory pressure continues to increase, growers are more likely to turn towards non-fumigant options, and (iii) cost of the new nematicides – with many growers facing increasingly shrinking margins, the price tag will be more important than ever. If the new nematicides are to replace fumigants, they will have to be integrated with a weed and soil disease management program, and such a strategy will have to provide comparable control at a similar cost than a fumigant program. There is probably no standard recipe for such a non-fumigant soil management program, as no fields are the same, and nematicides may work differently depending on soil and nematode type, and agronomic practice. Soil management programs will have to be more prescription-based and tailored towards the specific issues and needs of individual fields. Certainly, there are other advantages of moving away from fumigants and other more toxic nematicides, in terms of safety, public perception, and overall soil health. In the long-term, improved soil health and more resilient soils may be one of the greatest benefits of moving away from soil fumigants and adopting more selective and safer nematicides.
